# Editorial: Inflammation and Reparative Process After Cardiac Injury

**DOI:** 10.3389/fcvm.2019.00162

**Published:** 2019-11-08

**Authors:** Jean-Sébastien Silvestre, Ziad Mallat

**Affiliations:** ^1^Université de Paris, PARCC, INSERM, Paris, France; ^2^Department of Medicine, University of Cambridge, Cambridge, United Kingdom

**Keywords:** inflammation, myocardial infarction, innate and adaptive immune response, ischemia, therapeutic targets, heart failure

Despite innovative approaches in the treatment of acute cardiovascular events as well as efficient interventions in primary and secondary prevention, cardiovascular diseases remain the leading cause of death in industrialized countries and are expected to become so in emerging countries by 2020. Coronary artery disease (CAD) and its associated ischemic heart failure carry high mortality and morbidity. Intensive experimental and clinical research performed during the last few years has shed light on the instrumental role of innate and adaptive immunity in the cardiac response to ischemic injury.

The present series of reviews on this Research Topic summarizes and discusses the mounting evidence that the inflammatory response guides the reparative process following cardiac damage and that therapeutic modulation of specific inflammatory pathways could substantially enhance the efficiency of conventional treatment approaches in patients with cardiovascular diseases.

Innate and adaptive immunity coordinate distinct but mutually non-exclusive events governing cardiac repair. Among the actors of innate immunity, macrophages, whether originating from circulating monocytes or from the resident cardiac pool, appear to be major effectors of cardiac repair through their phagocytic capacity to clear damaged tissue and subsequent potential to promote inflammation resolution. In this context, Zhang et al. underline the decisive role of cardiac macrophage polarization from pro-inflammatory to inflammation-resolving phenotypes. In particular, they suggest that cellular metabolism governs macrophage-dependent function and integrates phagocytic activity with alteration of the transcriptional and epigenetic landscapes. Puhl and Steffens focus on neutrophils, some poorly studied actors in this context. They emphasize their ability to regulate reparative processes and, in particular, suggest that different neutrophil subsets could exist, which could exhibit distinct functional properties in the ischemic cardiac tissue.

The mechanisms governing cardiac repair likely involve multiple cellular and molecular interactions. In this line of reasoning, Psarras et al. discuss the interplay of innate immune cells with cardiac fibroblasts and cardiomyocytes as an integral component of the repair process following cardiac insult. Hanna and Frangogiannis review our current knowledge on the patterns and mechanisms of regulation and activation of TGF-β superfamily members in the infarcted heart, and discuss their cellular actions and downstream signaling mechanisms. Potere et al. recapitulate the cellular and molecular bases of Low-density lipoprotein receptor-related protein 1 (LRP1) functions in modulating the inflammatory reaction and the reparative process after injury in various peripheral tissues, and discuss recent evidences implicating LRP1 in myocardial inflammation and infarct healing. Recent studies identified intriguing roles for adaptive immune responses mediated by dendritic cells, T and B lymphocytes in myocardial remodeling following coronary artery occlusion. Santos-Zas et al. review this basic and experimental knowledge, which is already being tested in clinical trials.

Finally, Ferrini et al. tackle the emerging important field of biomedical engineering and its therapeutic applications in cardiac repair after injury. More particularly, they review the great potential of bioengineering strategies to improve the delivery and efficacy of immunomodulatory bioactive molecules, providing innovative and effective options to substantially alter the causative mechanisms underlying cardiac dysfunction.

We believe that this topic of cardio-immunology allows readers to fully appreciate the importance of the various molecular and cellular interactions underlying the bi-directional dialogue between inflammatory and non-inflammatory resident cells in the cardiac tissue. Targeting these critical biological pathways holds great promise for curbing the substantial burden of cardiovascular disease.

## Author Contributions

J-SS and ZM wrote this editorial and approved the manuscript.

### Conflict of Interest

The authors declare that the research was conducted in the absence of any commercial or financial relationships that could be construed as a potential conflict of interest.

